# Prediction of compound-target interactions of natural products using large-scale drug and protein information

**DOI:** 10.1186/s12859-016-1081-y

**Published:** 2016-07-28

**Authors:** Jongsoo Keum, Sunyong Yoo, Doheon Lee, Hojung Nam

**Affiliations:** 1 School of Electrical Engineering and Computer Science, Gwangju Institute of Science and Technology, 123 Cheomdangwagi-ro, Buk-gu, Gwangju, Republic of Korea; 2 Department of Bio and Brain Engineering, Korea Advanced Institute of Science and Technology (KAIST), Daejeon, 305-701 Republic of Korea

## Abstract

**Background:**

Verifying the proteins that are targeted by compounds of natural herbs will be helpful to select natural herb-based drug candidates. However, this entails a great deal of effort to clarify the interaction throughout in vitro or in vivo experiments. In this light, *in silico* prediction of the interactions between compounds and target proteins can help ease the efforts.

**Results:**

In this study, we performed *in silico* predictions of herbal compound target identification. First, data related to compounds, target proteins, and interactions between them are taken from the DrugBank database. Then we characterized six classes of compound-target interaction in humans including G-protein-coupled receptors (GPCRs), ion channel, enzymes, receptors, transporters, and other proteins. Also, classification-prediction models that predict the interactions between compounds and target proteins through a machine learning method were constructed using these matrices. As a result, AUC values of six classes are 0.94, 0.93, 0.90, 0.89, 0.91, and 0.76 respectively. Finally, the interactions of compounds from natural products were predicted using the constructed classification models. Furthermore, from our predicted results, we confirmed that several important disease related proteins were predicted as targets of natural herbal compounds.

**Conclusions:**

We constructed classification-prediction models that predict the interactions between compounds and target proteins. The constructed models showed good prediction performances, and numbers of potential natural compounds target proteins were predicted from our results.

## Background

The efficacy of the medicinal use of natural products dates back thousands of years. In more recent years, compounds derived from natural products have shown promising effects in drug discovery and drug development. For example, oseltamivir (trade name, Tamiflu), an antiviral medication used to treat influenza A and influenza B, is synthesized from shikimic acid, a naturally occurring substance found in Chinese star anise herb [1]. However, the detailed mechanism of action, including the target proteins of compounds, is known for just a few natural products. Moreover, identifying compound-target interactions through in vitro or in vivo experiments requires considerable efforts. In this regard, accurate *in silico* screening methods are necessary to predict interaction between compounds and target proteins.

Numerous studies on the prediction of interactions between compounds and target proteins have been reported. Yamanishi et al. implemented a systematic study on the prediction of compound-target protein interactions [2]. They suggested that the interaction can be predicted by using the structural similarity of compounds and the genomic sequence similarity. They computed the sequence similarities between proteins using normalized Smith-Waterman scores and the structural similarities between compounds using SIMCOMP, a graph-based method for comparing chemical structures [3, 4]. With respect to prediction methods, Belakley et al. provided a useful method, referred to as the bipartite local model (BLM), to accurately predict compound-target protein interactions [5]. BLM predicts target proteins of a given protein using the structural similarity of compounds, genomic similarity, and information of interactions between compounds and targets. Since this method shows promising performance in drug-target prediction, we adopted this method in our study to predict the interactions between herbal compounds and target proteins.

**Fig. 1:**
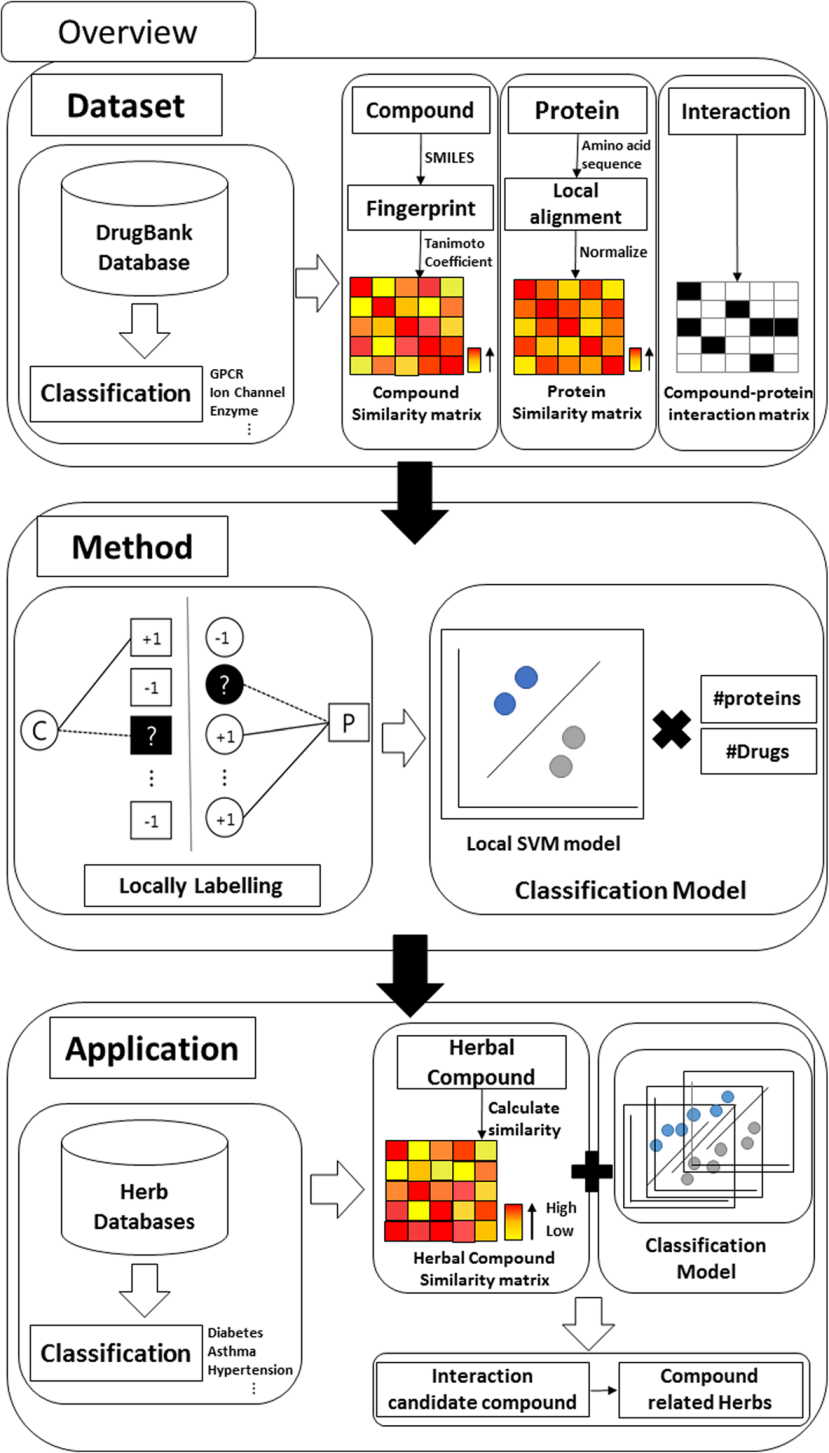
Overview of this study. First, compounds, target proteins, and the interactions between them are taken from the DrugBank database. These data are then classified into 6 types. After each similarity matrix is constructed, bipartite local models are made for predicting interactions using these matrices. Lastly, herbal compounds and target protein interactions are predicted by using the model

In this work, we constructed prediction models for interactions between compounds and target protein (Fig. 1). First, compounds, target proteins, and interactions thereof are taken from the DrugBank database [6–9]. These data are then classified into six types: G-protein-coupled receptors (GPCRs), enzymes, transporters, receptors, and other proteins. Next, compound structure similarity matrices of each type are calculated by using the Open Babel fingerprint (FP2). Genomic sequence similarity matrices of each type are calculated by using the Smith-Waterman algorithm and binary interaction matrices of each type are made using information of interactions between compounds and target proteins [4, 10]. After this process, bipartite local models are made for predicting interactions between compound and target proteins using these matrices. Lastly, herb data are taken from databases that have information on herbs such as TCMID, TCM-ID [11, 12] and KTKP (http://www.koreantk.com), and KAMPO (http://www.kampo.ca). Compounds of herbs and training data structural similarity matrices of each type are then calculated by using Open Babel [10]. By using these matrices and the bipartite local models, the herb-target protein interactions are predicted.

## Method

### Compound, target protein, interaction data

Most data related to compounds, target proteins, and interactions between them are taken from DrugBank database [6–9]. Then using IUPHAR/BPS Guide to PHARMACOLOGY database, these data are classified into six types, enzyme, GPCRs, transporter, ion channel, etc [13]. Table 1 shows the number of compounds, target proteins, and their interactions of each types. In our study, the number of compounds targeting enzymes, GPCRs, ion channels, other receptors, transporters, and other proteins is, respectively, 2107, 502, 311, 199, 410, and 83. The number of target proteins in these types is 404, 104, 117, 85, 95, and 26, respectively. The number of known interactions in these types is 2836, 1694, 1071, 587, 367, and 100, respectively. In this process, we use a compound that targets human organism. We then remove the data that do not have necessary properties such as the SMILES of the compound or the amino sequence of target protein. In the DrugBank database, proteins are classified into four types, target, enzyme, carrier, and transporter. Compounds only bind to proteins belonging to the target type as an inhibitor or an activator. We focus on targeting interactions such as inhibitors or activators rather than metabolic interactions such as substrates. Therefore, only proteins belonging to the target type are used in our study.

**Table 1:** The number of compounds, target proteins, and interactions of each types to construct a predicting model

	Compound	Target protein	Interaction
GPCR	502	104	1694
Ion Chanel	311	117	1071
Transporter	199	95	367
Receptor	410	85	587
Enzyme	2107	404	2836
Others	83	26	100

### Chemical similarity

Chemical structures can be identified by Simplified Molecular Input Line Entry Specification (SMILES). DrugBank database provides SMILES of each compound [8]. The structure similarity between two compounds is computed by using Open Babel, which provides fingerprints such as FP2, FP3, FP4, and MACCS from SMILES [10]. In this study, FP2, a path-based fingerprint that indexes small molecule fragments based on linear segments of up to seven atoms, is used. The similarity between the compound C_A_ and C_B_ is computed by using the formula (1).

1 $$ \mathrm{Tanimoto}\ {\mathrm{coefficient}}_{A,B} = \frac{AB}{A+B- AB} $$

*where AB : the number of bits set in molecules A and B*

*A: the number of bits set in molecules A, B : the number of bits set in molecules B*

The Tanimoto coefficient uses the bits set in both fingerprints. Applying the work to all compound pairs, the compound similarity matrix S_C_ is constructed.

### Genomic similarity

DrugBank database provides amino acid sequences of the target proteins [8]. In order to obtain similarities between the target proteins, we use the SIMD Smith-Waterman C++ Library, which calculates an amino sequence alignment score by using the Smith-Waterman algorithm [14]. The Smith-Waterman algorithm produces the optimal pairwise alignment between two sequences [4]. We then normalize the Smith-Waterman score. The normalized Smith-Waterman score between the protein P_A_ and P_B_ is computed by the formula (2)

2 $$ {S}_p\left({P}_A,{P}_B\right)=\frac{SW\left({P}_A,{P}_B\right)}{\sqrt{SW\left({P}_A,{P}_A\right)\times \sqrt{SW\left({P}_B,{P}_B\right)}}} $$

*where SW is the Smith – Waterman alignment score*

Applying this approach to all target protein pairs, the target protein genomic similarity matrix SP is constructed.

### Herbal compound data

Information of herbs and herbal compounds is taken from databases that have information of herbs such as TCMID, TCM-ID [11, 12] and KTKP and KAMPO. In this study, we use the herbs of which phenotypes are diabetes mellitus, hypertension and asthma. These phenotypes are selected because they are common diseases [15, 16]. Since SMILES of herbal compounds in these databases is ambiguous and has duplication, the SMILES of each compounds of herbs is obtained by using ChemSpider [17]. In this process, compounds that have ambiguous SMILES or do not have SMILES are removed and duplicated compounds are unified. Finally, the number of herbs that are related to diabetes mellitus, hypertension and asthma is 33, 93, and 251, respectively. The number of unique herbal compounds in these phenotypes is, respectively, 1303, 1720, and 3297. Table 2 shows the number of herbs and their compounds of each phenotype. The similarities between compounds of each herb and compounds of training data are then calculated by using Open Babel to predict the target protein by using the similarity between compounds and training data.

**Table 2:** The number of herbs and their compounds for predicting their target proteins. All data are classified by phenotypes

Phenotype	#Herb	#Compound (unique)
Diabetes	33	1303
Hypertension	93	1720
Asthma	251	3297

### Bipartite local model

**Fig. 2:**
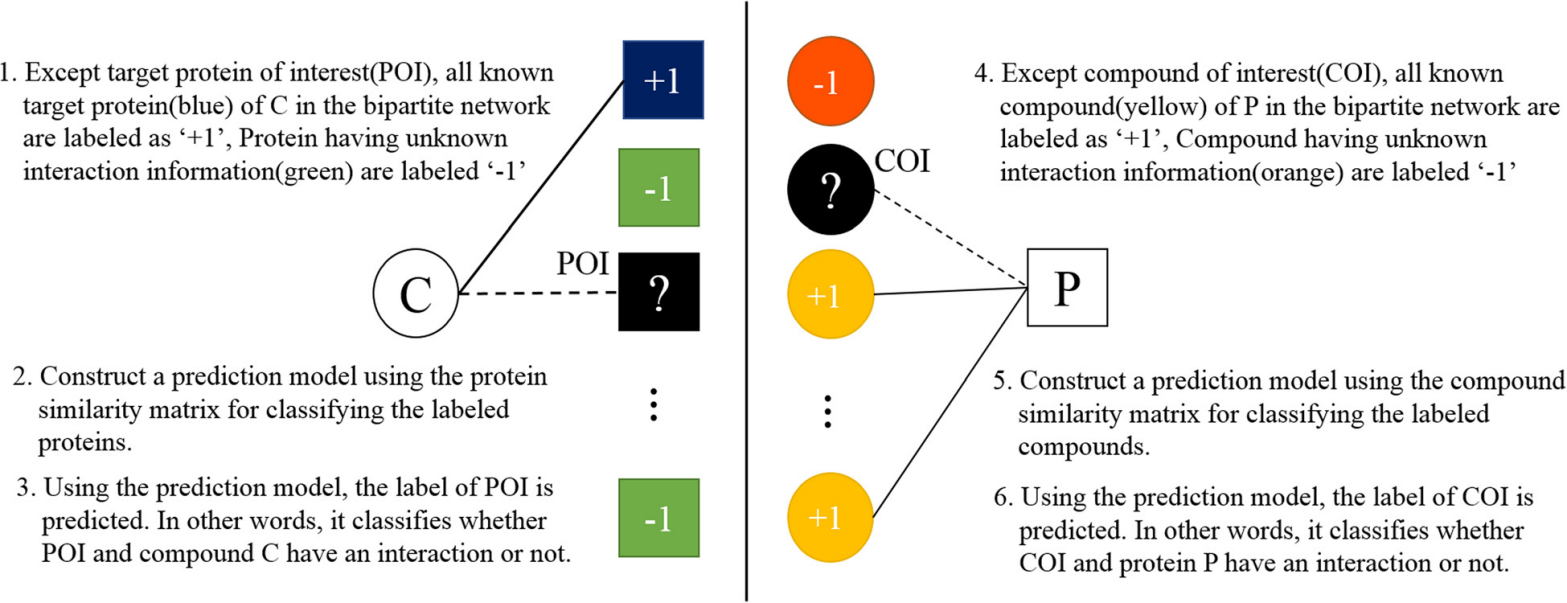
An illustration of protein prediction procedures (*left*) and compound prediction procedures (*right*)

In order to predict new interaction between compounds and targets, we use the Bipartite Local Model method [5]. In this method, compound-target protein interactions are represented by a bipartite graph. It predicts whether a compound C targets protein of interest (POI) or not and whether a protein P is targeted by compound of interest (COI) or not in the following Fig. 2.

### Support Vector Machines (SVMs)

Support vector machines (SVMs) are used as the classifiers for the bipartite local model. A support vector machine is a supervised learning model, used in classification and regression. The SVM classifies two classes with the maximum margin. Therefore, when new data are classified, it predicts more accurately than other methods. For this reason, SVMs are used for good performance of classification in many applications [18]. There are many libraries of available for SVM. The LIBSVM (v. 3.20) is used for the bipartite local model (BLM) [19]. Given similarity information about the vertices (either the compounds or the target proteins), each local SVM learns a function that can assign a continuous score to a compound or target from the labels of these vertices [5]. The sign of the score indicates the interaction or not. If the sign are positive, the predicted interactions between a compound and a target protein is positive, otherwise, they have no interactions. And the score contains the confidence of the prediction. If absolute value of the score is high, the predicted interaction is credible.

## Results

### Prediction performance

**Fig. 3:**
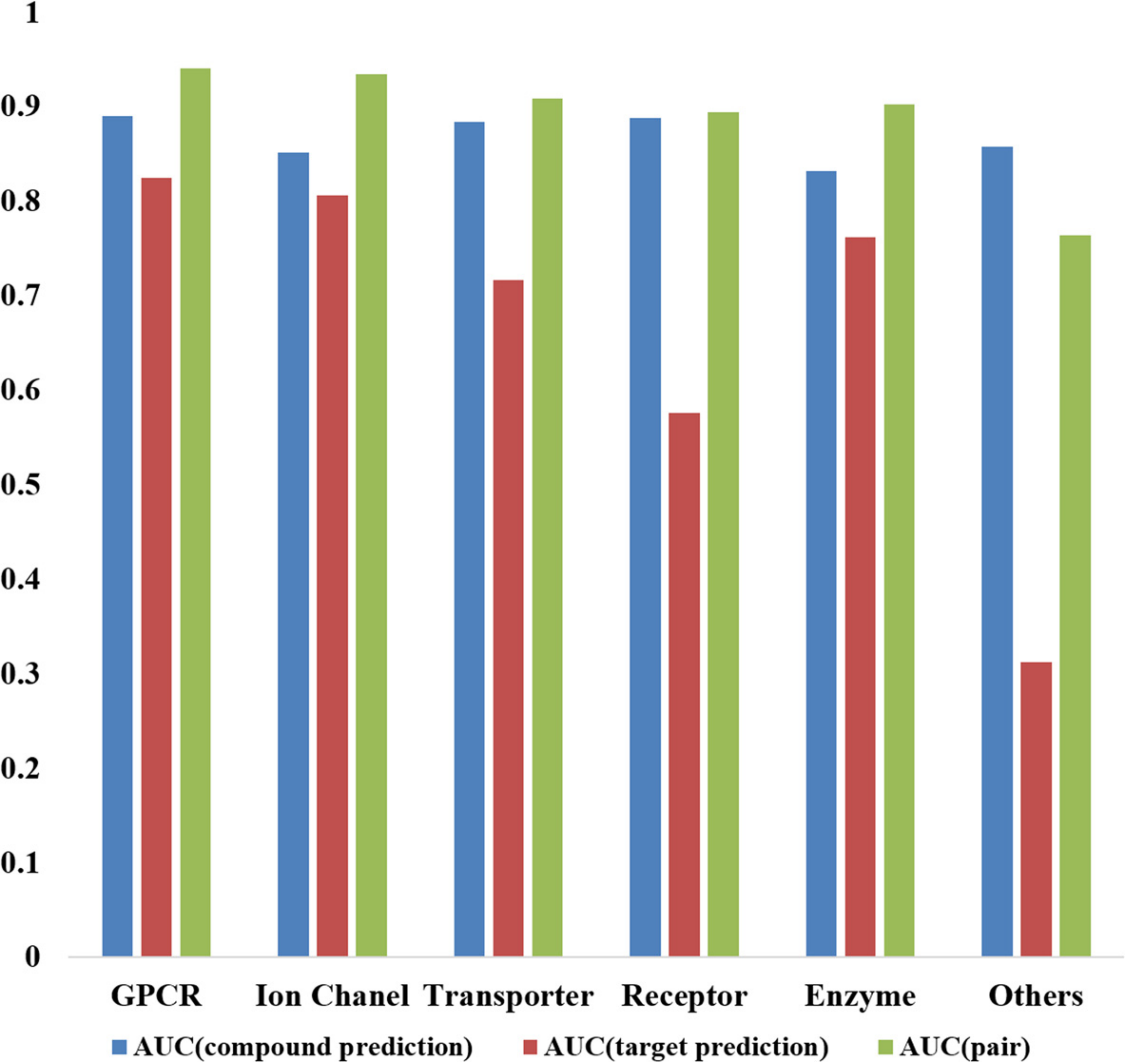
Performance of predicting model for each type of dataset

In order to estimate the performance of bipartite local model using our dataset, a 10-fold cross-validation is used [5]. For the reliable results, each dataset is randomly permuted. Then each of the datasets is divided into ten subsamples. After doing this, nine samples are used as a training dataset to make a classification model and the remaining one sample is used as a validation dataset for testing the model. The cross-validation process is repeated ten times, with each of the 10 subsamples used once as the validation data. Table 3 and Fig. 3 show the prediction performance for each type of dataset. Area under the ROC curve (AUC) and area under the precision-recall curve (AUPR) are used to evaluate the performance of the model. As the area is closer to one, the performance of the model is better. AUC (compound prediction) is evaluated by only using structural similarity matrix to predict which compounds target the proteins and AUC (protein prediction) is evaluated by only using genomic similarity matrix to predict proteins are targeted by the compounds. AUC (predicting compound and protein pair) and AUPR (predicting compound and protein pair) are evaluated by using both structural similarity matrix and genomic similarity matrix to predict more accurate interaction. The results show that the AUC of each type is about 0.9. Since this represents sufficiently high performance, the model can be used to predict interactions between the herb compounds and target proteins.

**Table 3:** Performance of predicting model for each type of dataset

	AUC (fixed target)	AUC (fixed drug)	AUC (pair)	AUPR (pair)
GPCR	0.8899	0.8246	0.9405	0.6274
Ion chanel	0.8514	0.8063	0.9339	0.6552
Transporter	0.8829	0.7163	0.9083	0.5768
Receptor	0.8877	0.5754	0.8932	0.5745
Enzyme	0.8315	0.761	0.9018	0.4337
Others	0.8574	0.3113	0.7634	0.4723

### Prediction results

**Fig. 4:**
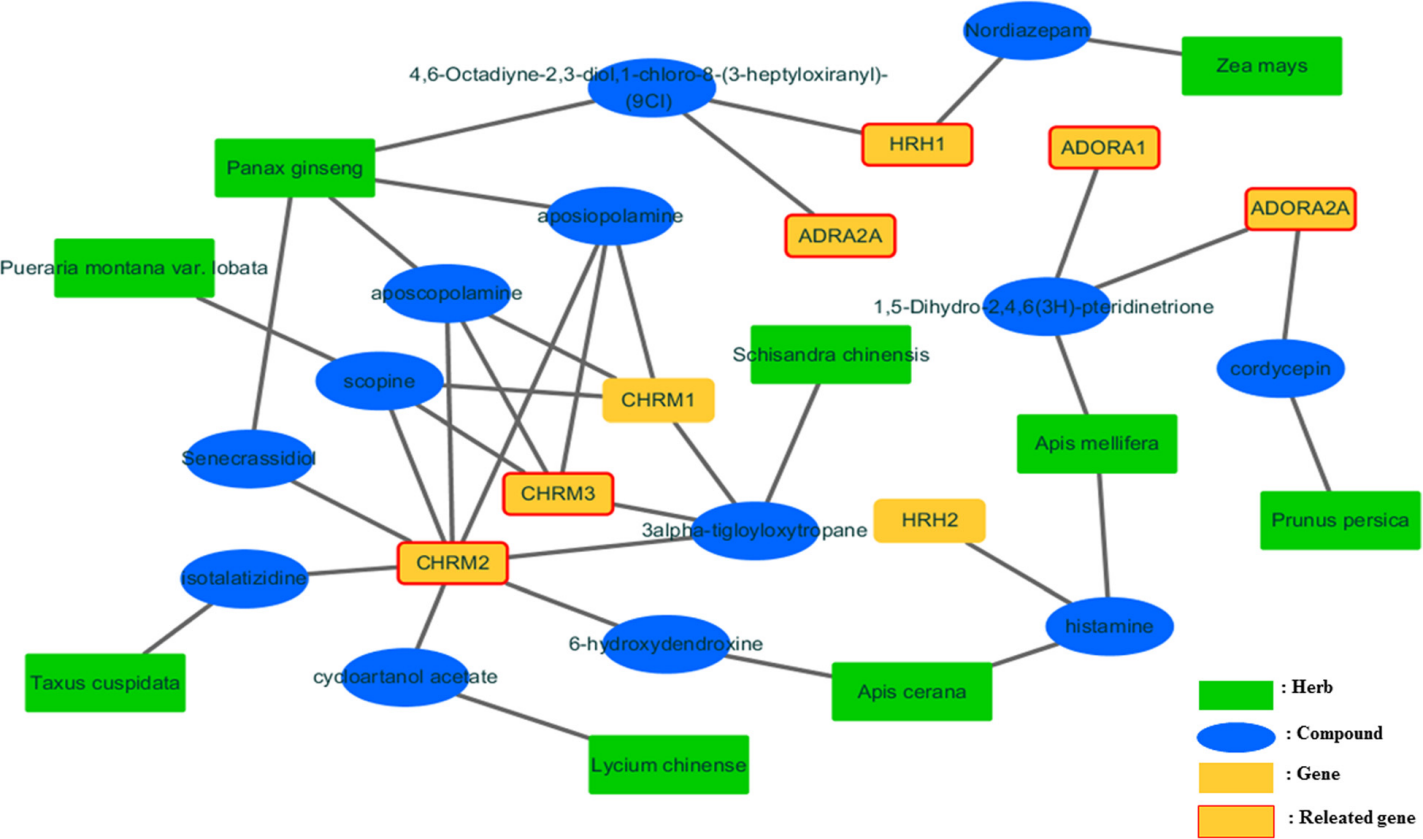
Predicted interaction network for the GPCRs data of diabetes mellitus phenotype. *Circles* indicate herbal compound, *rectangles* indicate herbs, and *rounded rectangles* indicate target proteins. *Rectangles* with a border represent target proteins that are related with diabetes mellitus

**Fig. 5:**
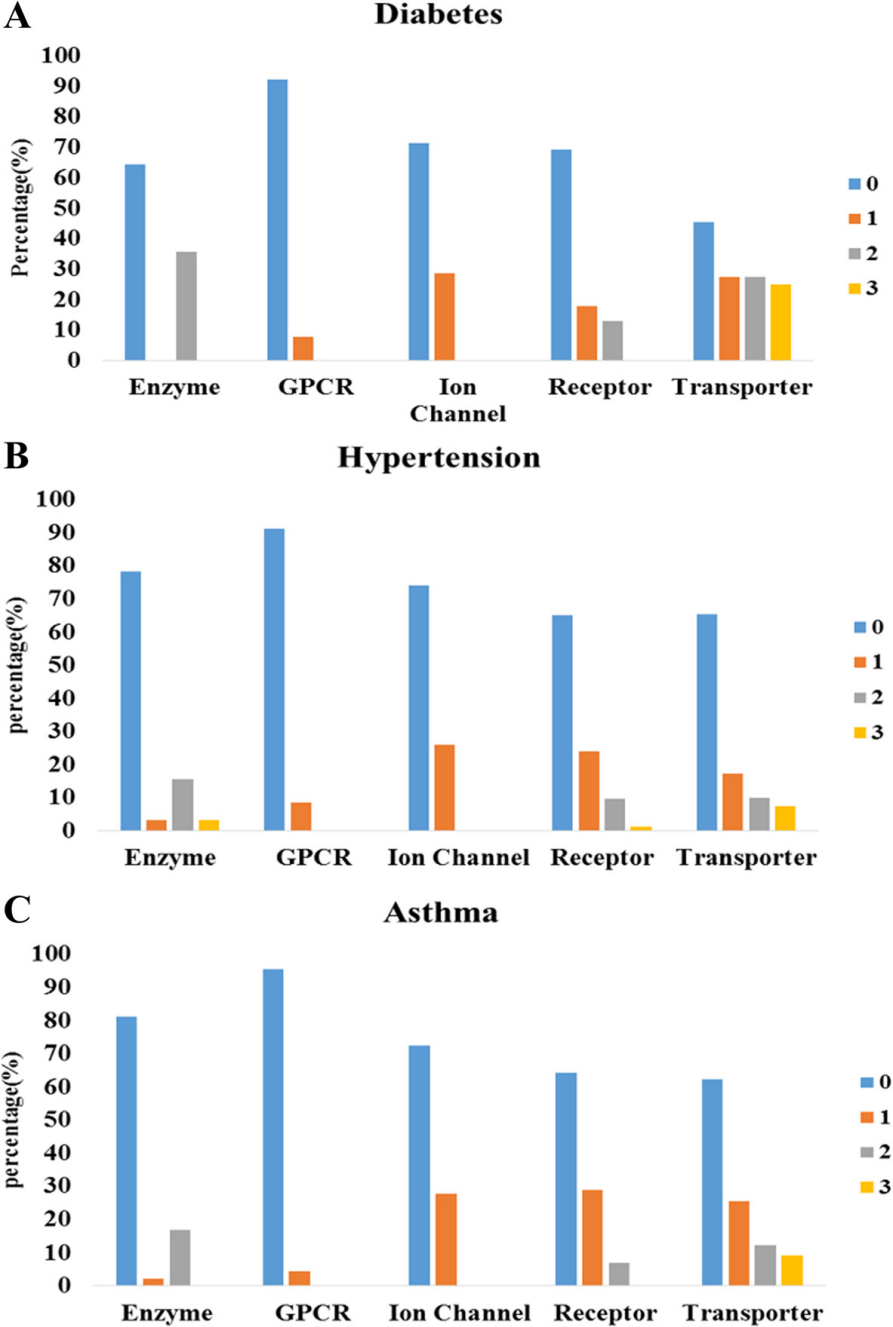
The proportion of Lipinski;s rule of five (ROF) of predicted herbal compound for each protein type in each phenotype. **a** The compound proportion of ROF in diabetes. **b** The compound proportion of ROF in hypertension. **c** The compound proportion of ROF in asthma. ROF is zero in most case

**Fig. 6:**
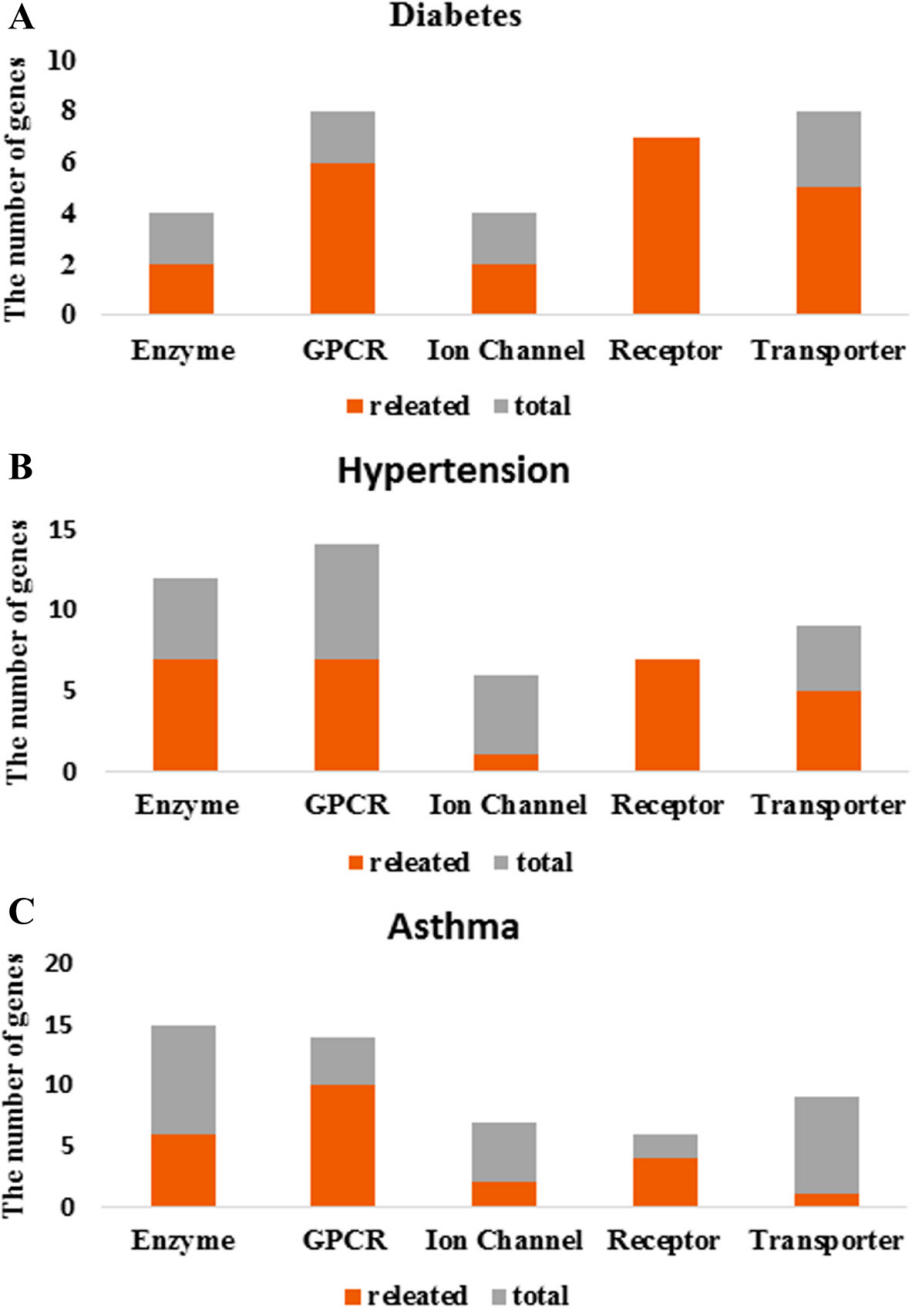
The number of predicted target proteins for each protein type in each phenotype. Part of the bottom indicates the number of target proteins that are related with each phenotype. **a** The number of predicted genes in diabetes. **b** The number of predicted genes in hypertension. **c** The number of predicted genes in asthma. Many proteins are related with each phenotype

In order to predict interactions between compounds of herbs and target proteins, the classification model is constructed using the training dataset. Since we focus on whether compounds bind to target proteins, the chemical similarity matrices alone are used to construct the prediction model. The prediction model is then used to predict whether herbs have an interaction or not. Table 4 summarizes the number of predicted herbs, their compounds, target proteins, and interactions between compounds and target proteins by protein type. All data are classified by phenotypes. In this results of asthma phenotype, the number of predicted herbs binding GPCRs, ion channels, transporters, other receptors, enzymes and other proteins is 38, 60, 127, 122, 57, and 11, respectively. The number of predicted compounds of herbs in these types is 44, 77, 275, 324, 48, and 10, respectively. The number of predicted target proteins in these types is 14, 7, 9, 6, 15, and 3 respectively. The number of predicted interactions between compounds and target proteins in these is 277, 237, 1055, 762, 268, and 29 respectively. Figure 4 is predicted interaction network for the GPCRs data of diabetes mellitus phenotype. Lipinski’s rule of five (ROF) of the predicted compound of each phenotype is then calculated by using its SMILES [20]. Figure 5 is graph that depicts a proportion of ROF of predicted herbal compounds for each protein types in each phenotype. The ROF of the compounds is zero in most cases. This result indicates that there are many compounds that can be used as drugs in herbs. We then find proteins that are related with asthma based on papers among proteins binding GPCRs, ion channels, transporters, other receptors, and enzymes. Figure 6 shows the number of predicted target proteins of each protein type in each phenotype. Part of the bottom indicates the number of target proteins that are related with each phenotype. In Fig. 4, we can show that many proteins are related with diabetes mellitus. The number of predicted target proteins related to asthma in papers is 6, 10, 2, 4, and 1, respectively. These results indicate that a substantial, number of predicted proteins that are targeted by herbs to treat asthma are related with asthma. This implies that the prediction model predicts well and proteins that are not related with asthma can be candidates to have a relation with asthma.

**Table 4:** The number of predicted herbs, their compounds, target proteins, and interactions by protein types. All data are classified by phenotypes

Phenotype	Type	#Herb	#Compound	#Gene	#Interaction
Diabetes	GPCR	9	13	8	24
	Ion Chanel	9	22	4	26
	Transporter	18	110	8	166
	Receptor	19	101	7	124
	Enzyme	8	14	4	21
	Other	1	1	1	1
Hypertension	GPCR	24	23	14	57
	Ion Chanel	28	52	6	99
	Transporter	47	121	9	286
	Receptor	46	174	7	296
	Enzyme	19	32	12	108
	Other	3	8	2	16
Asthma	GPCR	38	44	14	277
	Ion Chanel	60	77	7	237
	Transporter	127	275	9	1055
	Receptor	122	324	6	762
	Other	11	10	3	29

## Conclusions and discussion

In this research, we predict whether compounds bind to target proteins or not by using chemical structure similarity information and genomic sequence similarity information on a large scale. Most data that help to construct the predication model are taken from the DrugBank database. The classification models that predict whether compounds bind to proteins of GPCRs, ion channels, transporters, other receptors, enzymes and other proteins is validated by a 10-fold cross validation. The AUC score of each prediction model is about 90 %, except the prediction model of other proteins. Since the number of data of other protein types is much smaller than that of other types, it is difficult to construct an accurate prediction model. Nonetheless, most of the prediction models are reliable. We can therefore predict the interactions of herbs and target proteins using prediction models. In the prediction results, there are many predicted herbal compounds of which Lipinski’s rule of five (ROF) is zero. This implies that the herbs can be used as drugs. Furthermore, many proteins are predicted to bind to herbal compounds of each phenotype. Also, many studies contend that some proteins among them are related with each phenotype. This implies that the suggested model predicts well. Moreover, there are not only the proteins that already identified their relationship with each phenotype by other studies but also the other proteins.

We note that limited number of target proteins were used in our predictions considering the whole size of the genes found in human [21]. For example, the number of target proteins in the enzymes type is 404 in our dataset whereas the total number of proteins in the enzyme type is about 2,700 in the human genome [2]. Therefore, there are rooms to improve the performance of the prediction models with the increased size of the training data set. Also, the prediction performance could be improved using additional information of compounds rather than the structure similarity only. There are many useful properties that can distinguish the compounds such as human intestinal absorption (HIA), blood-brain barrier (BBB), etc. Lastly, although some compounds are related with proteins, the bipartite local model (BLM) method regards an unknown compound-target protein interaction as a non-interaction. This can degrade performance of the prediction model. When these problems are addressed, we can construct better classification models that predict the interactions between target proteins and compounds.

### Ethics approval and consent to participate

Not applicable.

### Consent for publication

Not applicable.

### Availability of data and materials

The dataset for constructing prediction model is available in the DrugBank database (http://www.drugbank.ca, version 4.0). The herb dataset supporting the conclusions of this article is available in the TCMID (http://www.megabionet.org/tcmid/), TCM-ID (http://bidd.nus.edu.sg/group/TCMsite/Default.aspx), KTKP (http://www.koreantk.com), and KAMPO (http://www.kampo.ca).
